# Italian Men Tested for BRCA1/2 Mutation: Psychological Distress during 6-Month Follow-Up

**DOI:** 10.1155/2020/3987935

**Published:** 2020-01-31

**Authors:** F. Pellini, S. Mirandola, E. Granuzzo, S. Urbani, G. Piccinni Leopardi, G. P. Pollini

**Affiliations:** Oncologic Surgery Department, Complex Operative Unit of Breast Surgery-Breast Unit AOUI, Verona, Italy

## Abstract

*Introduction*. Male breast cancer (MBC) is a rare disease, whose main risk factor is genetic vulnerability. Despite care of men with MBC is modeled on care of women, men's experiences with the disease and concerns related to the status of genetic mutation carrier are unique. So far, little is known concerning the psychological impact in BRCA1/2 testing, especially with regard to specific subset of individuals, such as male subjects and the elderly.

**Methods:**

We assessed self-reported anxiety and depression levels in 26 male subjects presenting at Unit of Breast Surgery in Breast Unit of AOUI Verona (MBC patients, *n* = 7; high-risk unaffected subjects, *n* = 7; high-risk unaffected subjects,

**Results:**

Among the 17 unaffected men tested, 7 (41%) received a positive test (either BRCA1 or BRCA2 pathogenic variant) and 10 (59%) a negative test. Of the 9 MBC patients tested, only one subject received a positive test result. No significant differences were observed in mean scores, mean change from baseline to follow-up, either for those with *T*+ or T− test results. *Discussion*. Genetic testing for BRCA1/2 mutation was not associated in our sample with increased level of psychological distress as measured with HADS in a short-term evaluation.

## 1. Introduction

Male breast cancer (MBC) is a rare disease, comprising only 0.1% of all men cancers [1] and about 1% of all breast cancer [2], while female breast cancer (FBC) comprises 25% of all cancers in females [3].

MBC tends to be diagnosed in later life, at a more advanced stage, and is considered similar to late-onset (postmenopausal) FBC [4]. Genetic vulnerability is a shared risk for MBC and FBC, and the most relevant risk factors are a family history of BC and the presence of pathogenic variants of BRCA gene [4].

Despite care of men with MBC is labeled on women's care, men's experiences with the disease and concerns related to the status of genetic mutation carrier are unique [4, 5].

Several studies focused on the short- and long-term psychological impacts of breast cancer on women [4] and other forms of cancer in the male and female population [6]. Given the rarity of MBC, a thorough understanding of the psychological implications of this condition is still lacking. Two relevant English studies aimed at deepening the experience and the psychological impact of MBC, through combination of tools for assessing general distress and cancer-specific distress, such as the Hospital Anxiety and Depression Scale (HADS) [7, 8] and Impact of Event Scale (IES) [7, 8]. Body image changes, measured using the Body Image Scale (BIS) [7], and coping strategies had also been evaluated [7, 8]. Another study on MBC survivors reported that 8% and 5% of the sample met the standard criteria for anxiety and depression [5].

Since the introduction in clinical practice of genetic testing for BRCA1/2 gene mutations, there has been a rising interest in eventual psychological distress caused by testing [9]. So far, no definite conclusions emerged and there is still a need for empirical evidence concerning the psychological impact of BRCA1/2 testing, especially with regard to specific subset of individuals, such as male subjects and the elderly.

Considering the gradual increase in MBC incidence [1, 2], it seemed appropriate and perhaps a bit provocative, to focus our attention on men suffering from this condition or at high-risk of developing it. Moreover, psychological features related to genetic testing for BRCA1/2 mutations have never been investigated in a population of Italian males.

The aim of this retrospective study is to assess self-reported anxiety and depression levels in male subjects presenting at Unit of Breast Surgery in Breast Unit of AOUI Verona. We specifically examined the scores obtained by these subjects in the HADS questionnaire administered before the genetic testing for BRCA gene mutations and during a 6-month follow-up visit.

## 2. Materials and Methods

This is a retrospective analysis of all male patients presenting with MBC and high-risk not-affected men, between 01/11/2015 and 31/01/2018, at the Unit of Breast Surgery at Verona AOUI. In our retrospective study were enrolled male subjects with a personal or family history of MBC, male subjects with a family member carrier of BRCA1/2 gene mutation, male subjects with a close family member diagnosed with female breast cancer (FBC) at 45 or younger, male subjects with a close family member diagnosed with bilateral breast cancer at any age, male subjects with three or more close family members diagnosed with breast cancer at any age, and male subjects with a close family member diagnosed with pancreatic cancer or metastatic PCa at any age or family history of ovarian cancer, pancreatic cancer, aggressive PCa, or metastatic PCa. Female subjects, male subjects affected by benign breast tumors, and healthy men with a family history not suggestive of genetic mutations linked to breast cancer were excluded.

Clinicopathological, psychological, and genetic data were obtained from Breast Unit Database (Gecos, Cartelle2000, DataBreast), which collects patients' and high-risk subjects' clinical records since January 1992. No additional tests were performed.

We registered male subjects' following data: current age, age at breast cancer diagnosis, breast cancer stage and treatment, family history of breast, ovarian, or prostate cancer in firstdegree relatives and BRCA 1/2 mutation status (if available), and psychological distress (HADS, Hospital Anxiety and Depression Scale). For MBC patients, we also recorded alcohol consumption and smoking habits, comorbidities, and history of psychological symptoms.

Patients diagnosed with MBC and high-risk not-affected men were candidated to be examined by an expert geneticist, specifically dedicated to the study of hereditary breast, ovarian, and prostate cancer. During ambulatory interview, the geneticist drawn genealogical family trees based on participants' family history. The BRCA test was performed through a blood sample collected at the Brest Unit and sent to the Medical Laboratory Department, where it was validated by fluorimetry technique and multiplex polymerase chain reaction (PCR). BRCA1 and BRCA2 mutations were classified according to their potential functional effect as recorded in the Breast Cancer Information Core (BIC) database [10] as class, 5 pathogenic; class 4, likely pathogenic; class 3, uncertain; class 2, likely not pathogenic; and class 1, not pathogenic.

Participants' psychological distress was assessed through the HADS at the time of testing for BRCA1/2 gene mutation and during a 6-month follow-up visit [11]. This is a four-point 14-item self-report instrument to assess anxiety (seven items) and depression (seven items) in somatic, psychiatric, primary care patients and in the general population [12]. Each item is scored from 0 to 3, so that the maximum for each subscale is 21. Cutoff points were lower than 8 (within normal range), 8 to 10 (possible clinical cases), and ≥11 (clinical cases) for both scales, respectively [11]. The HADS was translated into Italian and validated by Costantini et al. in a sample of cancer inpatients [13]. They also showed the validity of the total score as a reliable measure of general distress [13], so that the Italian version can be used as a screening questionnaire for people at increased risk of developing psychological or psychiatric conditions.

All collected data were recorded in a Microsoft Office Excel spreadsheet.

Continuous variables are expressed as mean and standard deviation. When comparing two groups with normal distributions, we applied *t*-tests for independent and paired samples. Nonparametric tests were used when appropriate due to skewed distributions or low patient numbers. All statistical analyses were performed with IBM SPSS 10.0.

## 3. Results and Discussion

We collected data on 26 male subjects, who presented at our center between November 2015 and January 2018, were eligible for BRCA testing and received either a positive result (carriers) or a negative result (noncarriers).

The mean age of participants was 58 years (SD = 12), seven subjects were >65 years of age. Thirty-five per cent of the subjects tested had been treated for MBC, while sixty-five per cent were unaffected subjects with strong family history for breast cancer; among them, 5 had other cancer than MBC (prostate cancer).

All the nine subjects affected by MBC underwent modified radical mastectomy, before genetic testing. Among those with MBC, 3 subjects were diagnosed with early-stage MBC (Ia-Ib) and were administered ormonotherapy and 6 subjects were administered chemotherapy plus ormonotherapy due to advanced cancer stage at diagnosis.

From January 2015 to January 2018, 26 men (7 MBC patients and 19 high-risk not-affected men) were engaged in genetic counseling and testing at our center and completed the HADS questionnaire in paper format prior to genetic testing and during a 6-month follow-up visit. Baseline characteristics of participants are shown in Table 1.

Between those with available data, namely, MBC patients, only one subject reported history of psychological symptoms. Most of the MBC patients (66%) had significant medical issues, such as hypertension, diabetes mellitus, or other cardiovascular diseases. Participants received genetic testing results between 2 and 4 weeks after completion of baseline assessment and were engaged in a 6-month follow-up visit. 8 subjects (30.8%) were found to be carriers and 18 noncarriers of BRCA1/2 gene mutation. Among the 17 unaffected men tested, 7 (41%) received a positive test (either BRCA1 or BRCA2 pathogenic variant) and 10 (59%) a negative test. Of the 9 MBC patients tested, only one subject received a positive test result.

Self-rated levels of anxiety and depression were recorded with the HADS in 19 subjects, both prior to genetic testing and during follow-up visit 6 months after carrier status disclosure. 7 subjects failed to complete the HADS questionnaire after receiving genetic testing results, so only baseline assessment data were available.

The affected and unaffected groups were divided into two subgroups (T+ and T−) according to their test results. No significant differences were observed in mean scores, mean change from baseline to follow-up, either for those with T+ or T− test results. Because of this lack of significant differences, only the findings on psychological distress for the unaffected group are shown (Table 2).

In our sample, 5 men (19%) were scored as possible cases of anxiety disorder at baseline. These subjects obtained a nearly significant decrease in the anxiety score from 9 (SD 1.2) to 4.4 (SD 3.8) (*P*=0.06), after receiving a negative test.

No significant difference in the self-reported distress level as measured with the HADS was detected between adult and elderly subjects.

## 4. Discussion

The main finding of our study is that genetic testing, carried out in a sample of men either affected or at high-risk for MBC, was not associated with increased level of psychological distress as measured with the HADS in a short-term evaluation. Furthermore, our data show that receiving either a positive or a negative result does not affect the level of self-reported psychological distress in a sample of high-risk unaffected men. In our sample, though small, there were no significant differences between adults and the elderly.

A relevant role in MBC pathogenesis is played by genetic risk factors; many studies showed that 15–20% of male patients with BC have a family history of breast cancer, a higher percentage than what observed in women with BC (7%) [14–16]. Accordingly, among our patients was recorded a high percentage of positive family history of BC, i.e., 42.3%. Among genetic risk factors, BRCA 1/2 gene mutations are widely recognized as relevant in MBC susceptibility [17]. In our case series, BRCA gene testing was carried out following ASCO recommendations on BRCA testing [14] in 26 male subjects presenting at our department between January 2016 and December 2018. Among them, 7 were diagnosed with MBC and 19 were healthy high-risk men.

The finding that nearly 31% of participants received a positive test is not surprising because we specifically selected for oncogenetic counselling high-risk subjects, following the ASCO recommendations mentioned above [14]. In this way, we were able to obtain a BRCA gene mutational study of some high-risk family clusters and therefore to involve these subjects in personalized screening programs, which include clinical breast examination, mammography, and contrast-enhanced MRI yearly, as well as clinical urologic examination, serum PSA level testing, and transrectal US for prostate evaluation yearly.

Numerous studies claim that, although to a lesser extent than women, men also expresse a certain degree of psychological distress linked to neoplastic disease [5]. Indeed, despite small sample size, our results show significantly higher mean scores on HADS-A and HADS-D in the affected than in the unaffected group. This result is in accordance with that reported by Reichelt et al. in a retrospective study conducted in 287 Norwegian women [19]. Ruddy et al. evaluated quality of life and symptoms in 42 MBC subjects and implemented the HADS for psychological distress assessment [5]. They found a 40% prevalence of abnormal scores, with 32% in HADS-A and 8% in HADS-D [5]. This finding is much better than ours, but it must be considered that our sample consists mainly of high-risk healthy subjects, which achieved lower average scores compared to affected subjects.

Both men with and without a personal history of MBC showed stable levels of depression and anxiety, measured by the HADS. These scores were either lower than or comparable to those of normative samples [18]. The finding of a sample mean lower than the normative mean of depressive symptoms could be attributed partly to the fact that the normative data used in this setting were derived from populations of a different nationality than the Italian one, for which normative data are not currently available. An alternative explanation could be that members of families in which there have been numerous cases of neoplastic diseases, develop resilience to stress and a positive elaboration of the concept of illness. Another alternative may be that the counselling and care they had received made them feel safe and that they believed the surveillance programs could provide them and their relatives with the best prevention and care strategies.

We found no significant variation in HADS-A and HADS-D scores from baseline to follow-up in men without cancer receiving either positive or negative BRCA1/2 test results. This finding seems to support the hypothesis that, in this population, test results do not influence the level of distress to any significant degree in short-term evaluation. This result is concordant with that of Reichelt et al. [19], who suggested that stability in mean scores of HADS in Norwegian women indicates a lack of significant traumatization in relation to genetic testing results. Schwartz et al. reported a significant reduction in psychological distress in women tested negative for BRCA1/2 mutation [20]; in our study, genetic testing did not appear to influence HADS scores very much. This difference could be to some extent imputed to the normal/below normal levels of distress at baseline recorded in our sample.

Our results support the main conclusions of other studies carried out in women, namely, that no adverse psychological consequences seem to arise from genetic testing for BRCA1/2 mutation [21, 22]. This hypothesis may, with increasing clinical evidence, also be valid in male subjects.

This study has several limitations. Firstly, the small sample size does not allow an absolute generalization of the observed results. Secondly, the absence of short-term increase in anxiety and depression levels does not guarantee the absence of deterioration in long-term psychological conditions. Lastly, we believe that psychological evaluation in a genetic-testing setting is a complex topic, which should be carried out using diversified and numerous tools to grasp the facets of individual experiences.

Further investigations are clearly needed in order to deepen knowledge upon psychological implication of genetic testing in male suffering from and at high-risk for MBC. We intend to expand the experience of our center by implementing the use of quantitative methods with focus groups and with the involvement of family members in psychological support interviews.

## Figures and Tables

**Table 1:** Baseline characteristics of MBC subjects and unaffected men in our sample.

	Unaffected men (*n* = 17)	MBC subjects (*n* = 9)
	Mean (SD)	Mean (SD) or frequency (%)
Age (years)	51.8 (13.2)^*∗*^	60.7 (7.0)^*∗*^
HADS-A	3.5 (3.1)^*∗∗*^	5.7 (3.1)^*∗∗*^
HADS-D	2 (2.0)^*∗*^	3.5 (1.9)^*∗*^
Alcohol consumption^1^	N/A	2 (22%)
Smoking status	N/A	4 (44%)
Psychological symptoms	N/A	1 (11%)
Relevant comorbidities	N/A	6 (66%)
Other cancer	N/A	1 (11%)

^*∗*^ *P* < 0.05; ^*∗∗*^*P*=0.05. ^1^>20 g/day.

**Table 2:** Baseline and follow-up scores of men without cancer, receiving positive and negative BRCA1/2 test results.

	Men without cancer, negative test (*n* = 10)		Men without cancer, positive test (*n* = 7)	
	Baseline Mean (SD)	Follow-up Mean (SD)	Baseline Mean (SD)	Follow-up Mean (SD)
HADS-A	3.4 (3.2)	1.6 (2.2)	3.6 (3.3)	5 (0.5)
HADS-D	2.3 (2.4)	2.1 (2.3)	1.6 (1.5)	1.5 (1.7)

## Data Availability

The data used to support the findings of this study are available from the corresponding author upon request.
